# Design new epoxy nanocomposite coatings based on metal vanadium oxy-phosphate M_0.5_VOPO_4_ for anti-corrosion applications

**DOI:** 10.1038/s41598-021-87567-3

**Published:** 2021-04-14

**Authors:** M. A. Deyab, Brahim El Bali, Q. Mohsen, Rachid Essehli

**Affiliations:** 1 Egyptian Petroleum Research Institute (EPRI), Nasr City, Cairo Egypt; 2 Independent scientists, Oujda, Morocco; 3 Department of Chemistry, College of Sciences, Taif University, Taif, Saudi Arabia; 4 Energy and Transportation Science Division, Oak Ridge National Laboratory, Oak Ridge, TN USA

**Keywords:** Chemistry, Corrosion

## Abstract

Epoxy nanocomposite coatings are an essential way to protect petroleum storage tanks from corrosion. For this purpose, the new nanocomposite epoxy coatings (P-M/epoxy composites) have been successfully designed. The P-M/epoxy composites are based on the metal vanadium oxy-phosphate M_0.5_VOPO_4_ (where M = Mg, Ni, and Zn). The function of P-M/epoxy composites as anti-corrosion coatings was explored using electrochemical and mechanical tests. Using electrochemical impedance spectroscopy (EIS), it has been noticed that the pore resistance and polarization resistance of the P-M/epoxy composites remain higher as compared to the neat epoxy. The P-M/epoxy composites have the greatest impact on the cathodic dis-bonded area and water absorption. Besides, P-M/epoxy composites exhibit a very high order of mechanical properties. Further, Mg_0.5_VOPO_4_ has the greatest effect on the anti-corrosion properties of epoxy coating followed by Zn_0.5_VOPO_4_ and Ni_0.5_VOPO_4_. All these properties lead to developing effective anti-corrosion coatings. Thus, the net result from this research work is highly promising and provides a potential for future works on the anti-corrosion coating.

## Introduction

The corrosion in the petroleum storage tanks causes heavy economical and environmental damages in the petroleum field^1–6^. The tank coatings are considered the first bumper wall to protect storage tanks from corrosion^7–9^. Despite this fact, many coatings suffer from several faults such as weak adhesion and high permeability^10–12^.

Nanocomposite coatings have received worthy concern for future using as anti-corrosion barriers for petroleum storage tanks. As we know, epoxy resin exhibits high anti-corrosion and mechanical properties than that of other resin material^13–15^. Though epoxy resin displays better anti-corrosion properties, it still lacks enough barrier layer owed to the high resin permeability, which leads to hinder the anti-corrosion performance^16^.

Indeed, nano-phosphate materials are essential components in nanocomposite epoxy coatings due to their ability to recover the epoxy damages because of the corrosive solution^17–19^. However, we noted that there are very few works on the influence of phosphates materials on the anti-corrosion properties of the coating.

Tian et al.^17^ have shown that α-zirconium phosphate has a great role in increasing the anti-corrosion properties of phosphate coating. Morozov et al.^20^ reported that cerium particles and organophosphate groups improved the corrosion resistance of the epoxy coating. They indicated that the integration between cerium and phosphates leads to a synergistic anti-corrosion effect on both anodic and cathodic sites. Deayb et al.^21^ indicated that the permeability of the epoxy resin was significantly improved by the incorporation of hydrogen-phosphate particles into the epoxy matrix and this led to the creation of a good anti-corrosion coating layer.

Recently, there is an urgent need in developing the performance of epoxy resin using phosphate compounds to protect the metal structures from corrosion. According to this, we develop new epoxy nanocomposites based on new vanadium oxy-phosphate compounds M_0.5_VOPO_4_ (M = Mg, Ni, and Zn). In this work, we combine electrochemical and mechanical assays to investigate how the title compounds influence the anti-corrosion properties of epoxy coating nanocomposites.

## Experimental section

### Synthesis of metal vanadium oxy-phosphate compounds

M_0.5_VOPO_4_ (where M = Mg, Ni and Zn) compounds were successfully prepared using a single step via a solvo-thermal route without employing any heat treatments according to the previously reported procedures^22,23^. Stoichiometric mixtures of NH_4_VO_3_ (Aldrich, ≥ 99.99%), acetic acid CH_3_COOH (Aldrich, ≥ 99.99%), M(NO_3_)_2_·6H_2_O (Aldrich, ≥ 99%), and NH_4_H_2_PO_4_ (Aldrich, 99.99%) were used in the synthesis. First, NH_4_VO_3_ and acetic acid CH_3_COOH with a mole ratio of 1:1 were dissolved in 20 ml of H_2_O to form a clear green solution (Solution A). M(NO_3_)_2_·nH_2_O was added into solution A and then was stirred at 70 °C for 30 min. NH_4_H_2_PO_4_ was then dissolved in 10 ml of H_2_O (Solution B). After stirring at 70 °C for 30 min, solution B was added to solution A drop-wise to form a new mixture, which was stirred at 70 °C for an additional 1 h. The solution was finally poured in a 100 ml autoclave which was then heated at 200 °C for 24 h. After filtering the solution, the obtained green powder was dried at 100 °C for 12 h under vacuum.

### Preparation of nanocomposite coatings and coated electrodes

The nanocomposite coatings (i.e. P–Ni/epoxy, P–Zn/epoxy and P–Mg/epoxy nanocomposites) were prepared by blending epoxy resin (type Bisphenol-based—Ciba Co.), poly-amidoamine hardener (Arkema Co.), xylene and 1.0% of M_0.5_VOPO_4_ (where M = Mg, Ni and Zn). All the ingredients were homogenized using a speed mixer for 3.0 h. The final formula was grounded for 2.0 h to achieve adequate fineness.

Carbon steel sheets (from petroleum storage tank source) were utilized as coated working electrodes. The electrode dimension is 12 mm × 16 mm × 0.50 mm). The preparation of working electrodes before the coating was conducted using the standard method ASTM G1-03^24,25^. The film applicator was used to apply a very thin layer on the steel surface. The coated electrodes were placed in the oven at 333 K to get a complete cure coating surface. The coating micro-meter (Mitutoyo) was used to measure the coating layer thickness. It was approximately 38 ± 5 μm.

### Electrochemical and mechanical experiments

EIS measurements were used to explore the anti-corrosion performance of new nanocomposite coatings. The adequate 3-electrodes (i.e. working, calomel electrode (SCE) and Pt electrodes) glass cell was used for EIS measurements. All experiments were conducted using Potentiostat//Galvanostat system type Gill-AC-947.

The EIS experiments conditions are:

- Frequency range = 0.01 Hz to 100 kHz,
- Amplitude = 10 mV,
- Operation potential = open circuit potential (OCP),
- Immersion time = 7 days,
- Temperature = 303 K.

Water absorption (*Ø*%) of the nanocomposite coatings was calculated using the coating capacitance from EIS experiments at initial (*C*_0_) and after 7 days (*C*_t_) of the immersion time. Brasher–Kingsbury relation was used to get *Ø*% for different nanocomposite coatings^26,27^.

1 $$\varnothing = {\text{ log}}\left( {C_{{\text{t}}} /C_{0} } \right)/{\text{log}}\,\varepsilon_{{{\text{H2O}}}} . $$

Here ε_H2O_ is the dielectric constant of H_2_O (ε_H2O_ = 80).

The cathodic disbanding experiments were conducted according to ASTM G8-96(2019)^28^.

All the mechanical experiments (i.e. bend test, cross-cut adhesion, contact angle and impact resistance) were conducted in accordance with ASTM D522, ASTM D 3359-17, ASTM D7334, and ASTM D2794, respectively^29–32^.

Differential scanning calorimetry (DSC) and Glass Transition Temperature (Tg) measurements were recorded by DSC 3 METTLER TOLEDO (Heating rate = 20 °C/min—nitrogen flow rate = 20 ml/min).

## Results and discussion

### XRD pattern of M_0.5_VOPO_4_

**Figure 1:**
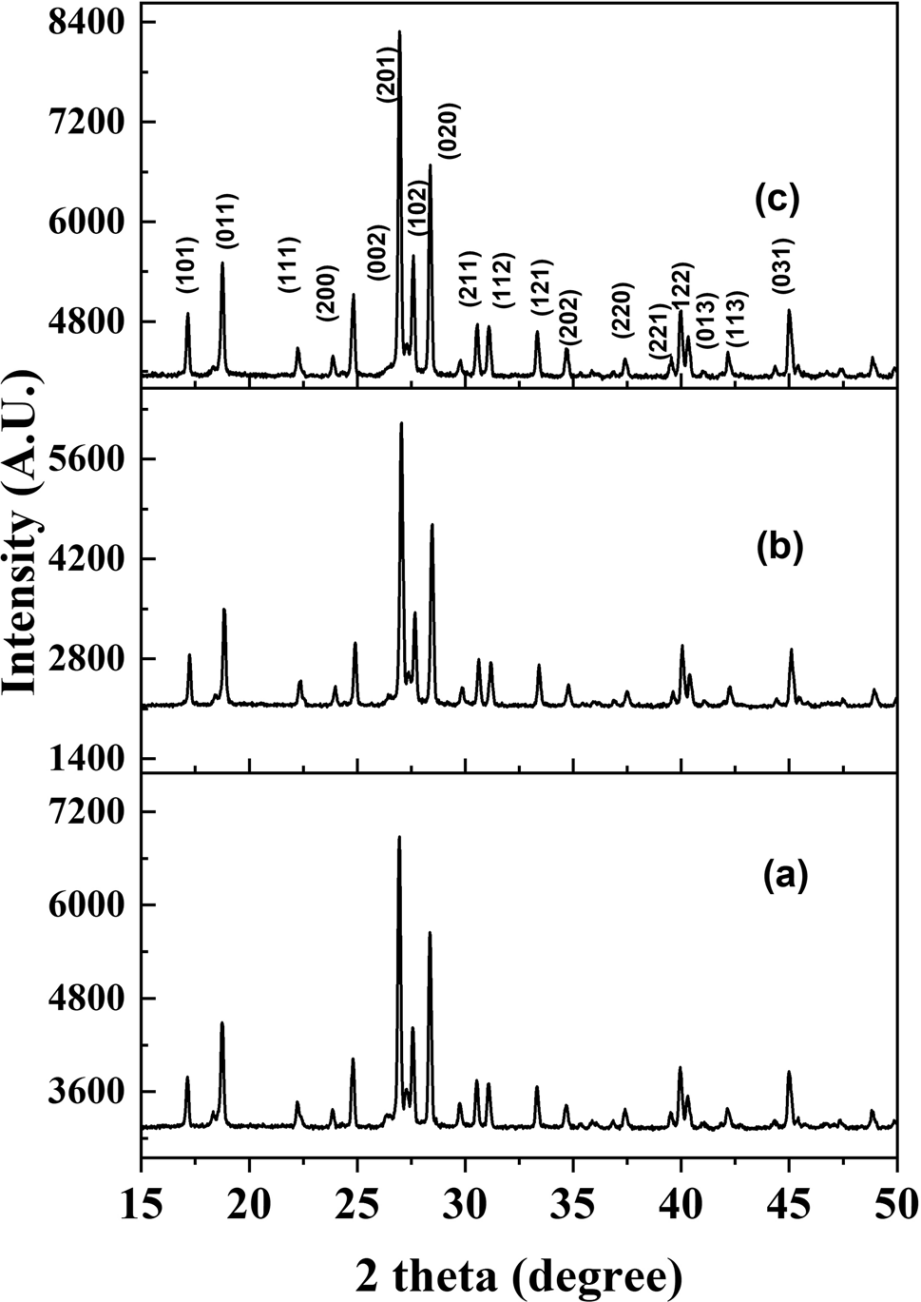
XRD pattern of M_0.5_VOPO_4_ (where M = Mg (**a**), Ni (**b**) and Zn (**c**)).

The M_0.5_VOPO_4_ (where M = Mg, Ni and Zn) compounds were analyzed using the powder X-Ray diffraction technique. The diffractogram of the materials was recorded in the 2-theta range of 10°–80° as illustrated in Fig. 1. The XRD pattern confirms the high purity of the synthesized M_0.5_VOPO_4_ materials without the presence of any crystallized impurities.

### Anti-corrosion properties of P-M/epoxy composites

**Figure 2:**
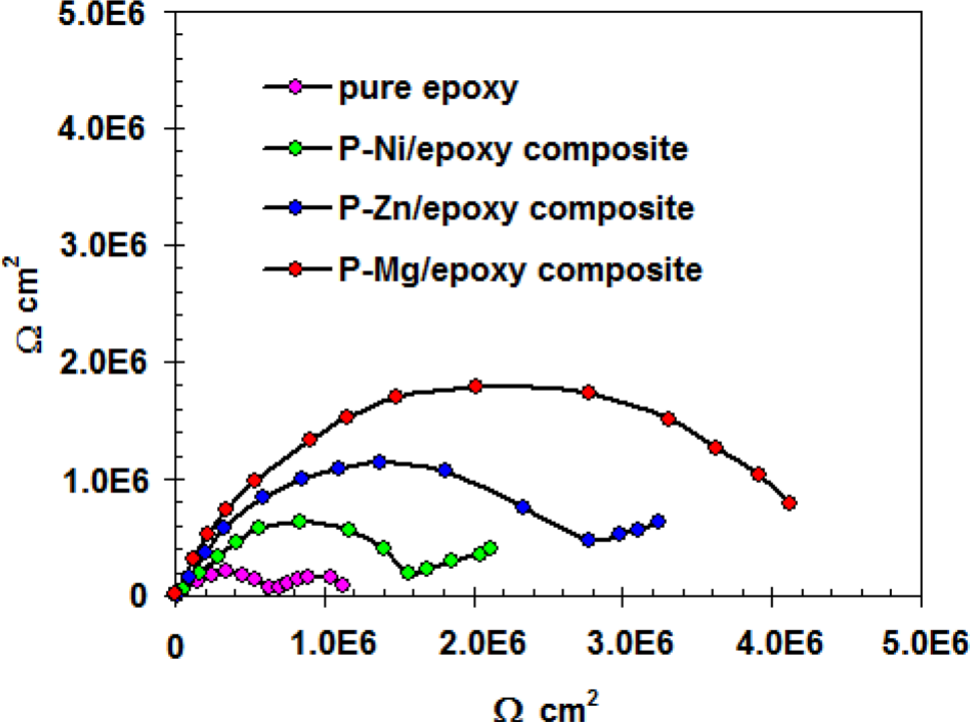
Nyquist plots for carbon steel electrodes coated with neat epoxy, P–Ni/epoxy nanocomposite, P–Zn/epoxy nanocomposite and P–Mg/epoxy nanocomposite in 3.5% NaCl solution at 303 K.

The influence of new synthesis phosphate compounds M_0.5_VOPO_4_ on the anti-corrosion properties of the epoxy coating was confirmed by the EIS studies. The Nyquist plots for carbon steel electrodes coated with neat epoxy, P–Ni/epoxy nanocomposite, P–Zn/epoxy nanocomposite and P–Mg/epoxy nanocomposite in 3.5% NaCl solution at 303 K are presented in Fig. 2. This figure demonstrates that the Nyquist plots for all coated electrodes have the two-time constants with the exception of P–Mg/epoxy nanocomposite which show one time constant. The appearance of a first peak at the high frequency for neat epoxy, P–Ni/epoxy nanocomposite and P–Zn/epoxy nanocomposite is attributed mainly to the coating layer^33^. While the second peak at the low frequency is due to the corrosion process under the coating layer^34^.

**Figure 3:**
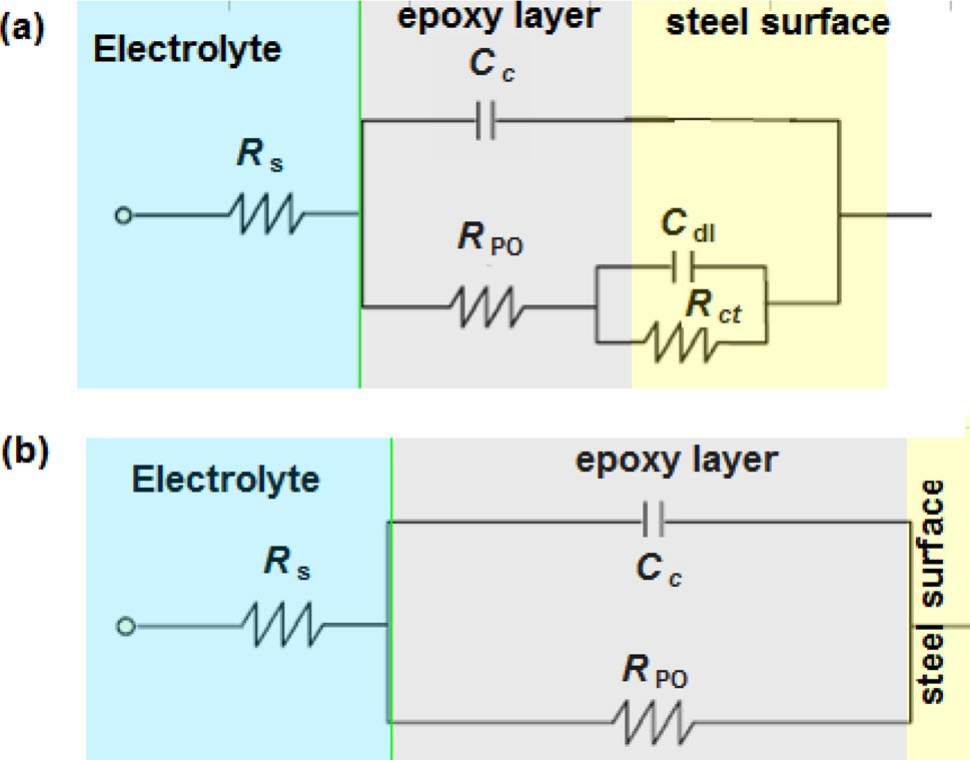
equivalent electric circuits for (**a**) neat epoxy, P–Ni/epoxy nanocomposite and P–Zn/epoxy nanocomposite; (**b**) P–Mg/epoxy composite.

Generally, most epoxy coatings deteriorate with time, causing more complicated impedance behavior than the excellent coating. Over time, the corrosive solution (i.e. 3.5% NaCl solution) penetrates the coating texture and forms solution/metal interface under the coating^35^. This leads to steel corrosion process at the liquid/metal interface (Fe(s) = Fe(aq)^2+^  + 2e)^36,37^. According to this situation, the most suitable equivalent electric circuit that fits the Nyquist plots for neat epoxy, P–Ni/epoxy nanocomposite and P–Zn/epoxy nanocomposite is shown in Fig. 3a. The elements of Fig. 3a are the capacitance of the epoxy coating (*C*_c_), pore resistance (*R*_po_), polarization resistance (*R*_p_), solution resistance (*R*_s_) and the capacitance of double layer (*C*_dl_). All these elements are listed in Table 1. We observed that both *R*_po_ and *R*_p_ values were significantly increased by using P–Ni/epoxy and P–Zn/epoxy composites comparing with *R*_po_ and *R*_p_ values in the case of pure epoxy. Interestingly, P–Mg/epoxy composite is able to heal the coating defect and form one time constant. In this case, the second peak at the low-frequency disappears (see Fig. 2) and the corresponding equivalent electric circuit is shown in Fig. 3b. Moreover, P–Mg/epoxy composite shows the highest *R*_po_ value (see Table 1).

**Table 1:** EIS parameters of coated carbon steel with neat epoxy and new nanocomposite coatings in 3.5% NaCl solution at 303 K.

System	*R*_po_ × 10^5^ Ω cm^2^	*C*_c_ × 10^–11^ F cm^−2^	*R*_ct_ × 10^5^ Ω cm^2^	*C*_dl_ × 10^–11^ F cm^−2^
Pure epoxy	6.38	4.30	4.65	5.56
P–Ni/epoxy composite	14.56	0.93	18.25	3.37
P–Zn/epoxy composite	22.49	0.64	25.16	0.83
P–Mg/epoxy composite	42.76	0.45	–	–

Following the same EIS data, it was clear that *C*_c_ and *C*_dl_ are linked to the barrier performance of composites coatings. The low *C*_c_ and *C*_dl_ values of new P-M/epoxy composites indicate their good barrier performance against corrosive solution^38,39^. It is worth noting that Mg_0.5_VOPO_4_ has the greatest effect on the anti-corrosion properties of epoxy coating followed by Zn_0.5_VOPO_4_ and Ni_0.5_VOPO_4_.

**Figure 4:**
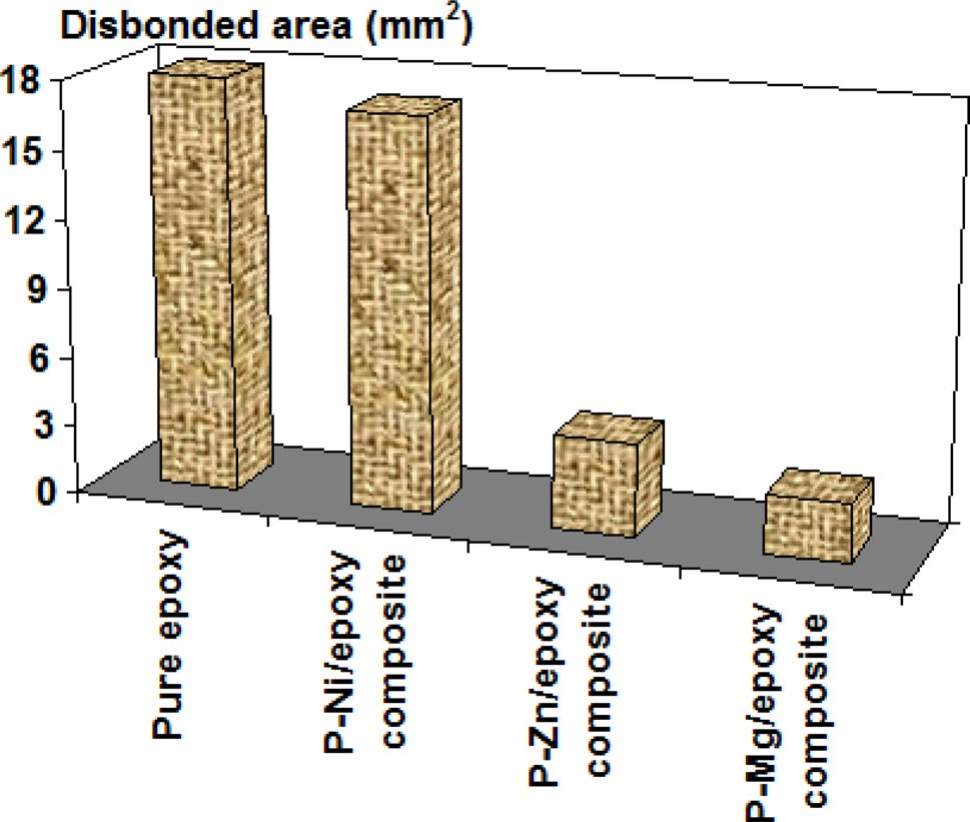
cathodic de-lamination data for neat epoxy, P–Ni/epoxy, P–Zn/epoxy and P–Mg/epoxy nanocomposites in 3.5% NaCl solution at 303 K.

The cathodic de-lamination tests for the new nanocomposite coatings are critical to investigate the strength of coatings adhesion with metal subtract^40^. The cathodic de-lamination data for neat epoxy, P–Ni/epoxy, P–Zn/epoxy and P–Mg/epoxy nanocomposites in 3.5% NaCl solution at 303 K are exhibited in Fig. 4. We noted that the incorporation of Ni_0.5_VOPO_4_ into the epoxy resin has a slight impact on the cathodic disbonded area. On the other side, the incorporation of Mg_0.5_VOPO_4_ and Zn_0.5_VOPO_4_ into the epoxy resin has a great impact on the cathodic disbonded area. This means that the new synthesis phosphate compounds could result in a strong adhesion between the epoxy resin and the steel substrate. Our study also confirms that Mg_0.5_VOPO_4_ has the greatest impact on the cathodic disbonded area followed by Zn_0.5_VOPO_4_.

**Figure 5:**
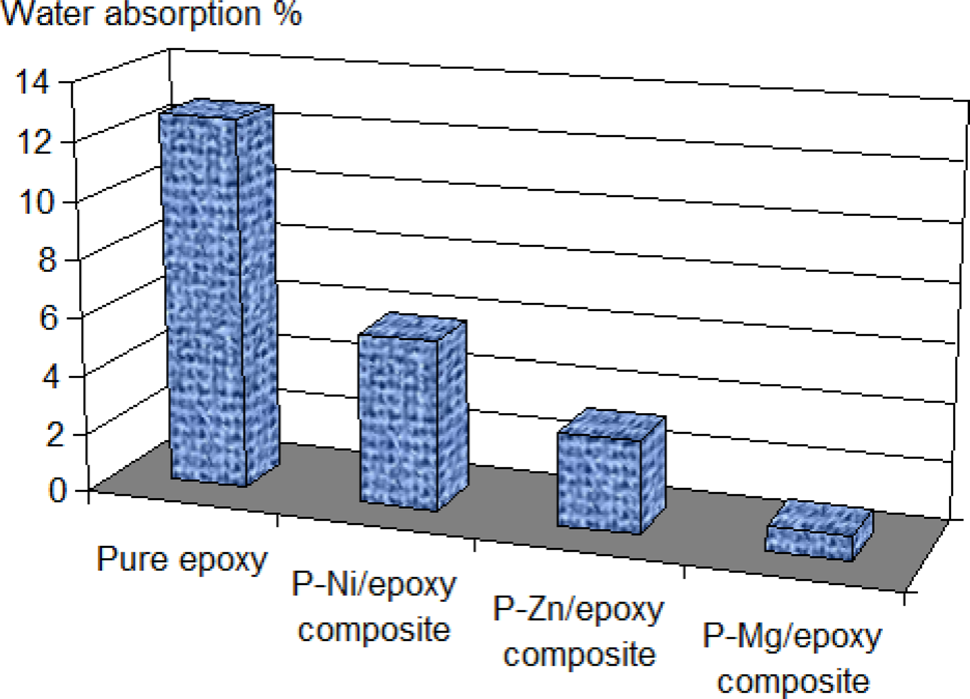
water absorption *Ø*% for neat epoxy, P–Ni/epoxy, P–Zn/epoxy and P–Mg/epoxy nanocomposites.

The electrolyte absorption by coating layer is the main factor in the quality of new coatings synthesis. Where the coating layer that absorbs less amount of corrosion electrolyte is characterized by a good barrier layer. According to this parameter, the results of the water absorption *Ø*% (see Eq. (1)) for neat epoxy, P–Ni/epoxy, P–Zn/epoxy and P–Mg/epoxy nanocomposites are presented in Fig. 5. In the case of neat epoxy, the water absorption *Ø*% was very high comparing with the P-M/epoxy composite. This indicates that the new P-M/epoxy composites are able to prevent the passage of the electrolyte inside the coating matrix. It is noting also that P–Mg/epoxy has the lowest *Ø*% followed by P–Zn/epoxy and P–Ni/epoxy. This confirms that Mg_0.5_VOPO_4_ plays a great role in the decline in water absorption by epoxy coating.

### Mechanical properties of P-M/epoxy composites

The mechanical tests (i.e. cross-cut adhesion, impact resistance, bend test and contact angle) further reveal the various mechanical features of epoxy coating acquired by incorporation by new synthesis phosphate compounds M_0.5_VOPO_4_. As illustrated in Table 2, in contrast to pure epoxy coating, cross-cut adhesion and bend tests are pass for all P**-**M/epoxy composites. We also observed a significant increase in the impact resistance of the coatings from 65 kg cm^−2^ in the case of pure epoxy to 85, 88 and 93 kg cm^−2^ in the cases of P–Ni/epoxy, P–Zn/epoxy and P–Mg/epoxy composites, respectively (see Table 2). This means that the presence of new synthesis phosphate compounds inside the epoxy matrix improves both the adhesion and the degree of the coating flexibility^41,42^. Moreover, the contact angle became wider with the addition of phosphate compounds from 61° in the case of pure epoxy to 88°, 89° and 89° in the cases of P–Ni/epoxy, P–Zn/epoxy and P–Mg/epoxy composites, respectively (see Table 2). The wider contact angles in the presence of phosphate compound mean that the new epoxy nanocomposites absorb less amount of corrosive solution, which confirms the anti-corrosion performance of new epoxy nanocomposites.

**Table 2:** Cross-cut adhesion, impact resistance, bend test and contact angle for coated carbon steel with neat epoxy and new nanocomposite coatings.

Coatings	Cross-cut adhesion	Impact resistance (kg cm^−2^)	Bend test	Contact angle (°)
Pure epoxy	1 mm fail	65	Pass	61
P–Ni/epoxy composite	1 mm pass	85	Pass	88
P–Zn/epoxy composite	1 mm pass	88	Pass	89
P–Mg/epoxy composite	1 mm pass	93	Pass	89

### The anticorrosive mechanism of P-M/epoxy composites

Epoxy coating permeability represents the vital defect in the coating layer leading to the failure in preventing the corrosive ions from transferring causing metal surface corrosion^43,44^.

Here, the incorporation of a small size of M_0.5_VOPO_4_ inside the epoxy matrix is able to heal the epoxy coating layer. According to the above data, the phosphate particles M_0.5_VOPO_4_ were distributed inside the pore of the epoxy matrix, leading to very low pore size. This action makes the zigzagging route for moving the corrosive ions is longer^45^, leading to the low possibility of the corrosion of steel surface and formation of iron oxide^46^.

**Figure 6:**
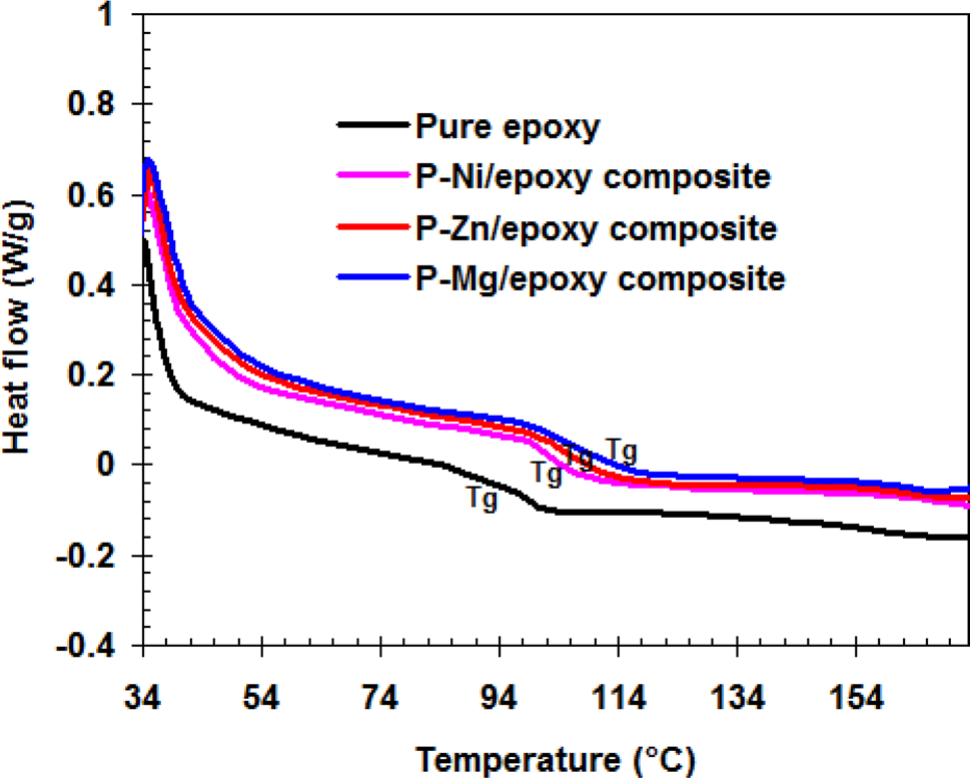
DSC thermograms for neat epoxy, P–Ni/epoxy, P–Zn/epoxy and P–Mg/epoxy nanocomposites.

The new epoxy nanocomposites are characterized by very good mechanical properties comparing with pure epoxy. The main reasons for this behavior are the improvement in the cross-linking of the epoxy matrix and the prevention in the epoxy layer disaggregation by the phosphate particles M_0.5_VOPO_4_^47^. DSC curves (see Fig. 6) support this statement. Where the incorporation of the small size of M_0.5_VOPO_4_ inside the epoxy matrix led to the increase in T_g_ from 91.4 °C for pure epoxy to 104.3 °C for P–Ni/epoxy, 105.7 °C for P–Zn/epoxy, and 107.2 °C for P–Mg/epoxy. This shifting in the T_g_ values is due to the increase in the cross-linking density of epoxy resin^48,49^. This behavior is responsible for the excellent mechanical properties of epoxy resin in the presence of phosphate particles M_0.5_VOPO_4_.

The type of metal atoms M = Mg, Ni and Zn in the structure of phosphate particles M_0.5_VOPO_4_ is the main factor in determining the anti-corrosion performance difference between new epoxy nanocomposites. Where the reduction electrode potential values of metal increase in the sequence: Mg < Zn < Ni^50^. Moreover, metals Mg and Zn have the ability to lose electrons more than iron atoms. This means that Mg and Zn can supply cathodic protection for steel surfaces. This leads to an additional anti-corrosion effect for epoxy nanocomposites besides their physical barrier against the corrosive electrolyte. On other hand, Ni is less active than the iron atom. This explains why P–Ni/epoxy nanocomposite is the lowest anti-corrosion performance. Also, Mg exhibits a very electronegative potential (i.e. − 1.75 V) comparing with Zn (− 1.1 V)^51^. This higher electronegative potential supplies more cathodic protection for steel surfaces resulting in higher anticorrosion properties.

## Conclusions

In this study, the new P-M/epoxy composites based on the vanadium oxy-phosphate M_0.5_VOPO_4_ (M = Mg, Ni and Zn) was successfully developed the anti-corrosion properties of epoxy coating nanocomposites were clearly investigated by electrochemical and mechanical measurements. In summary, the anti-corrosion properties of epoxy were improved by incorporating vanadophosphates inside the epoxy resin. This was clearly detected from the high values of pore resistance and polarization resistance. Our study also confirms that Mg_0.5_VOPO_4_ has the greatest impact on the cathodic disbonded area followed by Zn_0.5_VOPO_4_. The formation of epoxy nanocomposites containing vanadium oxy-phosphate was decisive for achieving excellent mechanical properties such as cross-cut adhesion, impact resistance, bend test and contact angle). The changing of the metal atoms M = Mg, Ni and Zn in the structure of M_0.5_VOPO_4_ particles is the main factor in determining the anti-corrosion performance difference between epoxy nanocomposites. This work establishes the great potential of the vanadophosphates/epoxy nanocomposites for the development of high-performance anti-corrosion coatings.
